# Association between glycemic status, renal function and emergency hospitalization for cardiovascular events in diabetes mellitus

**DOI:** 10.6026/973206300222859

**Published:** 2026-05-31

**Authors:** Syed Taqi Askari Shah, Toqeer Ahmad, Muhammad Ali Raza, Maryam Mushtaq, Waqas Ahmed, Reazun Nahar, Sakib Arsalan

**Affiliations:** 1 Department of Trauma and Orthopaedics, Queens Medical Centre, Nottingham University Hospitals, Nottingham, England; 2 Department of Acute Medicine, Junior Speciality Doctor, Midland Metropolitan University Hospital, Birmingham, England; 3 Department of Gastroenterology, DHQ Teaching Hospital Gujranwala, Gujranwala, Pakistan; 4 Department of Medicine, General Practitioner, Radiant Life Polyclinic, Dubai; 5 Department of Medicine, Medical Officer, Mayo Hospital Lahore, Lahore, Pakistan; 6 Department of Care of the Elderly, Medway Maritime Hospital, Gillingham, England

**Keywords:** Diabetes mellitus (DM), glycemic control, renal function, cardiovascular events, emergency hospitalization

## Abstract

Emergency hospitalization for cardiovascular events is a major clinical problem in patients with diabetes mellitus, especially when poor glycemic control and renal dysfunction coexist. Therefore, it is of interest to evaluate 225 diabetic patients presenting to the emergency department with cardiovascular symptoms, assessing glycemic status and renal function. Poor glycemic control (mean HbA1c 9.1 ± 1.8; 37.8% with HbA1c > 9%) and renal impairment (42.6% with eGFR < 60 mL/min/1.73m^2^) were common. Acute coronary syndrome (34.2%) was the most frequent presentation, with 72.4% requiring hospitalization and 21.7% ICU admission. HbA1c > 9% (adjusted OR = 2.34) and eGFR < [(60 mL/min)/1.73m^2^] (adjusted OR = 2.89) were independent predictors of emergency cardiovascular hospitalization.

## Background:

Diabetes mellitus has become one of the leading drivers of premature cardiovascular morbidity worldwide, shaping the burden of emergency hospitalizations in ways that modern health systems still struggle to manage effectively [1]. While the relationship between diabetes and cardiovascular disease is established, increasing evidence suggests that the likelihood of acute cardiovascular events is substantially influenced by two specific clinical factors: glycaemic control and renal function [2]. These two systems interact throughout the course of the disease and together, their deterioration can greatly increase the likelihood of unexpected hospital visits. Inefficient glycaemic control is the primary reason for cardiovascular instability. Endothelial dysfunction is induced by chronic hyperglycaemia and in addition, accelerates the formation of blood vessel plaques and increases their susceptibility to rupture through oxidative and inflammatory processes [3]. Endothelial dysfunction also increases the likelihood of cardiovascular events and numerous studies show the dose-response relationship between the increase in the HbA1c and that of myocardial infarction and acute coronary syndrome, heart failure (and its exacerbations) and arrhythmias [4]. The broad impact of diabetic nephropathy and renal function on cardiovascular health outcomes has been analysed. Diabetic nephropathy is one of the microvascular complications of diabetes and is also a cardiovascular risk multiplier. Early declines in kidney function cause loss of haemodynamic stability and cause an electrolyte imbalance, resulting in inflammation and a neurohormonal response, particularly the renin-angiotensin-aldosterone system [5]. This system, including the other supersystems, causes an overload of interstitial fluid, which is worsened by chronic pressure load and overload of fluid. This results in an exacerbated response to chronic pressure load, resulting in trabecular bulking, interstitial microvascular fluid, which is primarily calcified [6]. These changes in underlying chronic disease increase cardiovascular risk factors by presenting cardiovascular disease in an advanced and decompensated state [7]. Patients suffering from the combination of diabetes and chronic kidney disease often develop atypical and silent forms of cardiovascular disease which can also increase the risk of emergency disease presentation. These factors can all delay symptoms and make emergency care in advanced needs even more difficult [8]. Therefore, it is of interest to evaluate the association between glycemic status, renal function and emergency hospitalization for cardiovascular events in patients with diabetes mellitus.

## Methodology:

This study was conducted as a cross-sectional analytical study conducted at Mayo hospital, Lahore from March 2025 to August 2025. A total sample size of 225 patients was selected, calculated using the WHO sample size formula based on the expected prevalence of cardiovascular emergencies among diabetic individuals. A non-probability consecutive sampling technique was used, enrolling every eligible patient during the study period. Adults aged 18 years and above with type 1 or type 2 diabetes mellitus that presented with cardiovascular symptoms and had complete laboratory data for HbA1c, blood glucose ,serum creatinine and eGFR were included after providing informed consent. Patients were excluded if they had renal disease unrelated to diabetes, recent surgery or trauma, severe systemic infection such as sepsis, pregnancy, incomplete medical records, or if they left against medical advice.

## Data collection:

Following Institutional Review Board approval, diabetic patients presenting to the emergency department with cardiovascular symptoms were screened for eligibility and written informed consent was obtained. Data were collected using a structured proforma, including demographics, duration of diabetes, comorbidities and smoking status. Glycemic status was assessed through HbA1c and blood glucose levels, while renal function was evaluated using serum creatinine, eGFR and microalbuminuria when available. Cardiovascular presentations were documented based on physician-diagnosed conditions such as acute coronary syndrome, arrhythmias, hypertensive emergencies, acute heart failure and syncope. Hospitalization outcomes, including admission status, length of stay, ICU requirement and in-hospital complications, were also recorded. All laboratory results were retrieved from electronic medical records and patient confidentiality was ensured by anonymizing and securely storing the data.

## Data analysis:

Data were analyzed using SPSS version 26. Continuous variables such as age, HbA1c, serum creatinine and eGFR were summarized as mean ± standard deviation, while categorical variables, including cardiovascular diagnoses and hospitalization outcomes, were presented as frequencies and percentages. Associations between glycemic status, renal function and cardiovascular emergency hospitalization were assessed using Chi-square or Fisher's exact tests for categorical variables and an independent t-test for continuous variables, depending on normality of distribution. Correlation analyses using Pearson or Spearman coefficients were performed. A p-value of ≤0.05 was considered statistically significant.

## Results and Discussion:

Data were collected from 225 patients, average age was 58.6 ± 10.4 years, reflecting an older population with long-standing disease and males made up slightly more than half of the sample (54.2%). The mean duration of diabetes was 11.2 ± 6.3 years and most patients were overweight or obese, demonstrated by a mean BMI of 28.4 ± 4.1 kg/m^2^. Smoking history was present in 31.1% of the participants, while hypertension and dyslipidemia were highly prevalent at 69.7% and 52.4%, respectively, illustrating a heavy burden of cardiovascular risk factors. Glycemic status was notably poor, with a mean HbA1c of 9.1 ± 1.8% and over one-third of the patients (37.8%) had HbA1c levels above 9%. Hyperglycemia at presentation was also common, seen in 61.3% of cases. Renal impairment was frequent, with 42.6% showing eGFR < 60 mL/min/1.73m^2^ and 48.4% presenting with microalbuminuria or proteinuria, while 37.7% had creatinine ≥ 1.5 mg/dL, indicating significant chronic kidney disease in the cohort. In terms of cardiovascular presentations, acute coronary syndrome was the most frequent (34.2%), followed by hypertensive emergencies (22.7%), acute heart failure (18.6%), arrhythmias (14.2%) and syncope or presyncope (10.2%). A high proportion of patients (72.4%) required hospital admission and nearly one in five required ICU care (21.7%) (Table 1). The mean length of stay was 4.3 ± 2.1 days. Patients with HbA1c greater than 9% had more than double the odds of requiring emergency hospitalization (adjusted OR = 2.34), indicating that poor long-term glycemic control substantially increased cardiovascular risk. Impaired renal function, defined as eGFR < 60 mL/min/1. 73m^2^ , showed an even stronger association, with nearly a three-fold increase in hospitalization risk (adjusted OR = 2.89). Hyperglycemia at presentation was also a significant predictor, raising the likelihood of hospitalization by 1.78 times (Table 2).

This study evaluated the relationship between glycemic status, renal function and emergency hospitalization for cardiovascular events in patients with diabetes mellitus and the findings demonstrated a clear and clinically meaningful association between these parameters. The results indicated that more prominent features of the multidimensional cardiometabolic burden associated with diabetes (e.g., acute presentations of cardiovascular disease) are more likely when diabetes is combined with poor glycemic control, chronic poor glycemic control, renal impairment and prolonged disease duration. Most patients in this study had poor glycemic control, with an average baseline HbA1c of 9.1% and 33% or more of the study population had HbA1c levels above 9%. This is consistent with previous studies identifying a chronic persistence of HbA1c levels, associated with multiple end-organ disruptions (e.g., endothelium, plaques, autonomic nervous system and systemic inflammation) [9]. The data showed that patients with over 9% HbA1c were more likely to present with acute coronary syndrome, hypertensive crisis and acute heart failure. This is also consistent with literature that indicates that poor glycemic control is a chronic risk factor and along with unstable glycemic levels, becomes an acute risk factor to trigger cardiovascular events when there is portal systemic metabolic stress. Renal impairment was a strong risk factor for predicting cardiovascular emergency admissions [10]. About 41% of the study population had an altered eGFR and nearly 50% showed evidence of microalbuminuria and proteinuria. The contribution of declining kidney function to mechanisms of fluid overload, imbalanced electrolytes, vascular calcification and sympathetic overactivity, which contribute to the deterioration of the cardiovascular system, has been shown by other studies as well [11]. The risk of acute cardiovascular hospitalisation increases by almost four times as a result of combining hyperglycaemia and renal dysfunction. The synergistic effect is as other studies have shown in the metabolic-cardiorenal axis, associating the decline in renal function and glycemic dysfunction as the factors contributing to increased cardiovascular risk, along with the acceleration of nephropathy. The large number of patients with these abnormalities in this study shows the need to incorporate diabetes management in a multifaceted way, addressing glycemic control and protection of renal health [12]. The data on cardiovascular events of acute coronary syndrome, hypertensive crisis and acute heart failure are consistent with other studies on this population. This data further supports the claim that diabetes mellitus, to be managed at a population level, requires addressing both glycemic control and renal health to end poor health outcomes. The decline of renal health and glycemic control as system dysfunction are interrelated factors driving acute cardiovascular poor outcomes. This study emphasizes the importance of continuous monitoring of patients post-discharge and the identification of renal function decline before the implementation of significant interventions in order to mitigate the escalating pattern of inpatient cardiovascular comorbidity emergencies.

## Conclusion:

Poor glycemic control and impaired renal function were significant and independent predictors of emergency cardiovascular hospitalization among patients with diabetes mellitus. Individuals with elevated HbA1c levels and reduced eGFR were more likely to present with acute coronary syndrome, hypertensive emergencies, arrhythmias and acute heart failure, demonstrating the strong interplay between metabolic instability and renal deterioration in precipitating acute cardiovascular events.

## Advancement to knowledge:

This study shows that poor glycemic control and renal impairment independently predict emergency cardiovascular hospitalization in diabetic patients, supporting integrated cardiorenal-metabolic risk stratification in emergency practice.

## Figures and Tables

**Table 1:** Baseline demographic and clinical characteristics (n = 225)

**Variable**	**Mean ± SD / n (%)**
Age (years)	58.6 ± 10.4
Gender (Male)	122 (54.2%)
Gender (Female)	103 (45.8%)
Duration of Diabetes (years)	11.2 ± 6.3
BMI (kg/m^2^)	28.4 ± 4.1
Smoking (Current/Past)	70 (31.1%)
Hypertension	157 (69.7%)
Dyslipidemia	118 (52.4%)
**Glycemic Parameters**	
HbA1c (%)	9.1 ± 1.8
HbA1c < 7%	41 (18.2%)
HbA1c 7-9%	99 (44.0%)
HbA1c > 9%	85 (37.8%)
Random Blood Glucose (mg/dL)	268 ± 74
Hyperglycemia at Presentation	138 (61.3%)
**Renal Parameters**	
Serum Creatinine (mg/dL)	1.58 ± 0.62
eGFR < 60 mL/min/1.73m^2^	96 (42.6%)
Microalbuminuria/Proteinuria	109 (48.4%)
Creatinine ≥ 1.5 mg/dL	85 (37.7%)
**Cardiovascular Events**	
Acute Coronary Syndrome	77 (34.2%)
Hypertensive Emergency	51 (22.7%)
Acute Heart Failure	42 (18.6%)
Arrhythmias	32 (14.2%)
Syncope/Presyncope	23 (10.2%)
Required Hospital Admission	163 (72.4%)
ICU Admission	49 (21.7%)
Mean Length of Stay (days)	4.3 ± 2.1
In-hospital Complications	38 (16.9%)
Mortality	8 (3.5%)

**Table 2:** Logistic regression analysis for predictors of emergency cardiovascular hospitalization

**Predictor**	**Adjusted OR**	**95% CI**	**p-value**
HbA1c > 9%	2.34	1.42-3.82	<0.01
eGFR < 60 mL/min/1.73m^2^	2.89	1.76-4.72	<0.001
Hyperglycemia at Presentation	1.78	1.11-2.84	0.02
Duration of Diabetes > 10 years	1.41	0.90-2.22	0.11
